# The first record the *Limnia unguicornis* (Diptera, Sciomyzidae) parasites on a vulnerable pulmonate land snail, *Vertigo moulinsiana* (Gastropoda: Eupulmonata: Vertiginidae) and a literature review on *Limnia* species

**DOI:** 10.1007/s00436-024-08388-7

**Published:** 2024-11-01

**Authors:** Jacek Wendzonka, Urszula Sobczyńska, Zofia Książkiewicz

**Affiliations:** 1 Natural History Collections, Faculty of Biology, Adam Mickiewicz University, Uniwersytetu Poznańskiego 6, 61-614 Poznań, Poland; 2 Molecular Biology Techniques Laboratory, Faculty of Biology, Adam Mickiewicz University, Uniwersytetu Poznańskiego 6, 61-614 Poznań, Poland; 3 Department of General Zoology, Faculty of Biology, Adam Mickiewicz University, Uniwersytetu Poznańskiego 6, 61-614 Poznań, Poland

**Keywords:** Mollusca, Malacophagous flies, Parasitism, Life cycle, Threats, Wetlands, DNA analysis

## Abstract

The literature about mollusc-parasite interactions is focused on species affecting human health, such as trematodes on freshwater snails as intermediate hosts. Far less attention has been paid to parasite-snail interactions in terrestrial habitats. Here we present the first observation of a sciomyzid larva of *Limnia unguicornis*, parasitizing a vulnerable, tiny air-breading snail, *Vertigo moulinsiana* (strictly protected by Polish law). Sciomyzids are almost exclusively malacophagous but their biology and ecology are understudied. Thus, we have reviewed the available information on *Limnia* species and discussed the results of our research.

**Supplementary Information:**

The online version contains supplementary material available at 10.1007/s00436-024-08388-7.

## Introduction

Some gastropods are commonly known intermediate hosts for various parasite species described in the literature. Many of these interactions are discussed in the classroom already at an early education stage because they involve humans as final hosts. One of the best-known examples is a snail (usually *Galba truncatula*, among 22 lymnaeid species) as an intermediate host of a cosmopolitan trematode, *Fasciola hepatica* (Stewart 1999; Vázquez et al. 2018). This trematode is extremely dangerous for human health — the infestation may lead to liver damage, including cirrhosis (Machicado et al. 2016). A less horrifying example for human well-being is cercarial dermatitis caused by *Trichobilharzia szidati.* Its life cycle also involves lymnaeid snails, usually *Lymnaea stagnalis* (Macháček et al. 2018). Such examples of mollusc-parasite interactions are not rare. Numerous studies concern human-affecting schistosomiasis and freshwater snails as intermediate hosts (Giannelli et al. 2016). However, far less attention is paid to parasite-snail interactions in terrestrial habitats. One of the most spectacular relations occurs between a trematode, *Leucochloridium paradoxum*, and succinid snails in Europe and Asia (Nakao et al. 2019; Usmanova et al. 2023). Infestation by *L. paradoxum* results in an astonishing manifestation. The trematode’s larval form — cercaria — develops within the snail’s tentacle that is transformed into pulsating, green broodsacs, imitating a fat, tasty caterpillar. The trematode larva alters the host behaviour, making the snail more visible for predatory birds — the primary hosts (Bakke 1980; Wesołowska and Wesołowski 2013). Recently, also studies of parasite-snail interactions involving a giant land snail, *Lissachatina fulica*, have gained popularity. The snail is a widespread invader, a popular exotic pet, and a notorious vector of the rat lungworm, *Angiostrongylus cantonensis*, causing eosinophilic meningitis in humans (Gippet et al. 2023).

In this study, however, we focus on other parasite-snail interactions, unrelated to human health. These interactions are concealed and mysterious, involving a microcosm hidden from man and the drama covered within the snail shell. We aimed to take a closer look at an interplay between the Sciomyzidae (Diptera) and land snails. Sciomyzids are almost exclusively malacophagous. They occur in a great variety of habitats, from terrestrial to aquatic (Vala and Knutson 1990). According to the cited authors, larvae of these snail-killing flies are predators or parasitoids of molluscs. Although sciomyzid life cycles are well-studied, there are still many pending questions. Most of the doubts are related to instars, including the host selection process and how the larva invades a mollusc. Here we present the first record of sciomyzid larva parasitizing a vulnerable, tiny air-breading snail *Vertigo moulinsiana*, which is strictly protected by Polish law.

## Material and methods

### Snail collection and documentation

Thirty individuals of *V. moulinsiana* used for analyses were harvested in July 2021, for other purposes. The collection site is located in western Poland (geographical coordinates: 52.473582N, 16.792111′E; Palaearctic realm), at the edge of a lake and covered mostly with *Carex acutiformis* and *Phragmites australis*. Snails were found in boggy microhabitats, waterlogged for most of the year.

The individuals for dissections were collected manually, directly from monocot plants and immediately preserved in 96% alcohol. Shells and larvae were photographed and measured with an OLYMPUS SZX16 stereo microscope and OLYMPUS DP74 digital camera, using cellSens Dimension and Helicon Focus 8 software.

### Molecular analyses

#### DNA extraction

Total genomic DNA was extracted from the parasite specimen by using DNeasy Blood & Tissue Kit (Qiagen, Germany), according to the manufacturer’s protocol. In brief, the sample was incubated in 180 µl of ATL Buffer and 20 µl of Proteinase K (BioBasic, Canada) for 24 h at 56 °C with shaking at 600 rpm. DNA was eluted in 100 µl of 10 mM Tris pH 8.0.

#### Amplification of mini-COI for NGS sequencing

DNA barcode covering 322 bp from the 5′ end of the cytochrome c oxidase subunit I gene (COI) was amplified using primers bcdF01 (CATTTTCHACTAAYCATAARGATATTGG) (Dabert et al. 2010) and bcdR06 (GGDGGRTAHACAGTYCAHCCNGT) (Trzebny et al. 2020), tailed at 5′ ends with dual-indexed adapters (forward tail: CCATCTCATCCCTGCGTGTCTCCGACTCAG-index-GAT, reverse tail: CCTCTCTATGGGCAGTCGGTGAT-index) for sequencing using the Ion Torrent system (Life Technologies, USA). Polymerase chain reaction (PCR) amplification was performed in a 10-µl volume containing 1 × Hot FIREPol DNA Polymerase (Solis BioDyne, Estonia), 0.25 µM of each tailed primer and 2 µl of template DNA. Amplification program was as follows: 12 min at 95 °C, followed by 30 cycles of 15 s at 95 °C, 90 s at 50 °C and 30 s at 72 °C, with a final extension step at 72 °C for 5 min.

Since the parasite was isolated from host tissues, a next-generation sequencing approach was used for amplicon sequencing to eliminate possible host-derived sequence reads. Amplicons were purified using 2% E-Gel SizeSelect II Agarose Gels system (Invitrogen, USA), according to the manufacturer’s protocol. DNA concentration and fragment length distribution of the libraries were established using High Sensitivity D1000 Screen Tape assay on 2200 Tape Station system (Agilent Technologies, USA). Clonal template amplification and sequencing was performed on the Ion GeneStudio S5 System (Ion Torrent) using the Ion 540 Kit-OT2 chemistry and 540 Chip.

#### Read processing and data analysis

Bioinformatic analysis was conducted as reported by Trzebny et al. (2020, 2023). Briefly, quality filtered sequences were separated into individual combinations of indexes and trimmed at the 5′ and 3′ ends to exclude PCR primers. The sequences were then denoised to generate amplicon sequencing variants, using the DADA2 denoise-pyro method implemented in QIIME2 version 2023.5 (Bolyen et al. 2019; Callahan et al. 2016). The amplicon sequencing variants were compared to those in the GenBank using BLASTN (Morgulis et al. 2008; Zhang et al. 2000) with a 97% identity threshold to determine species.

## Parasite and host: species description

### Parasite

The family *Sciomyzidae* includes 541 species worldwide (Li et al. 2019), including 176 described from the Palearctic realm (Murphy et al. 2012). Life cycles of the vast majority of these dipterans are inextricably linked with terrestrial and freshwater gastropods. The larval stages pray on or parasite snails and slugs (Murphy et al. 2012). The biology of the *Sciomyzidae*, as compared to other dipteran families, is relatively well but unevenly studied. The life cycles were described for 240 species, while larvae were identified for 176 of them (Murphy et al. 2012).

The genus *Limnia* consists of 22 species: 5 in Palearctic realm and 17 in the Nearctic (Vala et al. 2012). Two species only are known from Poland: *Limnia unguicornis* (Scopoli, 1763) and *L. paludicola* Elberg, 1965. They are closely related (Tóthová et al. 2013) and difficult to distinguish by morphological characteristics (Rozkošný 1984; Vala and Knutson 1990). Their morphological similarity has led Vikhrev (2023) to a conclusion that *L. unguicornis* and *L. paludicola* are in fact one and the same species. The sciomyzid larva found by us parasitic in *Vertigo moulinsiana* is probably *L. unguicornis*. The applied genetic analyses give us 97% certainty, so only a very low uncertainty exists, also when considering the suggestion of Vikhrev (2023) that *L. unguicornis* should be synonymized with *L. paludicola.* Both species have a similar geographical range and may co-occur (Rozkošný 1984; Vala and Knutson 1990), but *L. unguicornis* is usually much more frequent and numerous. Therefore, the literature data and references cited by us in this text mostly concern *L. unguicornis*.

The biology of both species is understudied in many aspects, and much of the data come only from research conducted in laboratory conditions. *L. unguicornis* is a West-Palearctic animal (Rozkošný 1984) but its range includes Europe and Asia expanding to China (Li et al. 2019) and reaching southern fringes of Iran (Khaghaninia et al. 2016). It is probably the most numerous representative of the Sciomyzidae within its European range (Vala and Knutson 1990), including Poland (Kaczorowska 2007; Dubiel and Bystrowski 2019). The imago dwells in various habitats ranging from dry and sandy to moist and wet. Flights take place between May and October. Oviposition starts ca. 100 days after the flights begin. Eggs reach up to 1 mm in diameter and the morphology of larvae is well described (Vala and Knutson 1990). Three larval stages occur; the first larva hatches after 4–7 days and the third larva reaches about 10 mm in length (max. 12 mm). After the larval developmental period, lasting ca. 20–30 days, pupae in puparia (the fourth preimaginal stage) overwinter outside the host’s body (Vala and Knutson 1990). Therefore, *L. unguicornis* is a univoltine species.

Literature data concerning its host species are scarce. According to Beaver (1972), the first two larval stages of *L. unguicornis* have parasitoid habits, whereas the third-instar larva becomes a predator or a scavenger feeding on snails of the genera *Lymnea*, *Physa*, *Planorbis* and *Succinea*. Vala and Knutson (1990) observed feeding on *Succinea putris* (Linnaeus, 1758), *Lauria cylindracea* (da Costa, 1778), and *Deroceras reticulatum* (Muller, 1774) in laboratory cultures.

The biology of larvae is not fully recognized yet. Gaps in the knowledge concern the parasite’s attack strategy and host penetration. Vala and Knutson (1990) suggested that larvae usually mug the animals, which die due to their reduced motility. However, the process of host-selection remains a matter of speculation. *L. unguicornis* usually utilizes just one host individual during the entire larval developmental period, but rare events of host-switching also occur (Vala and Knutson 1990). According to the cited authors, it may happen when the primarily chosen snail (host) is too small to provide sufficient resources for larva to complete its development.

### Host

*Vertigo moulinsiana* is an Atlantic-Mediterranean species, with localities scattered mostly over the southern parts of Europe from the Pyrenees to Transcaucasia (Pokryszko 1990). Due to its population decline and shrinking range, *V. moulinsana* in the IUCN Red List of Threatened Species is classified as vulnerable (VU). The species is also listed in Annex II of the EU Habitat Directive and legally protected in some European countries (Pokryszko 2003). It is highly conservation-dependent and susceptible to many stress factors present in the lowland wetlands where it lives (Killeen et al. 2012).

The morphology of *V. moulinsiana* is rather inconspicuous from the human perspective. It is a tiny, dextral snail with a delicate, glossy, ovoid shell (shell height 2.25–2.73 mm and breadth: 1.33–1.65 mm; aperture height 0.65–0.83 mm and breadth: 0.88–1.08 mm) (Pokryszko 1990). Within the heart-shaped aperture, 4–9 teeth are present. Pokryszko (1997) listed several functions for the apertural barriers: (1) decreasing evaporation area; (2) obstruction against breach by small arthropod predators; and (3) mechanical reinforcement of fragile shell walls.

The vertiginid is a typical wetland species, usually associated with calcareous swamps (Killeen 2003a,b). It occurs where the water level is at the ground surface or very close to it for at least some of the year (Tattersfield and McInnes 2003). The close relationships found between the snail’s abundance and water level suggest that the site’s hydrology is a major factor determining the local distribution of the animal (Killeen 2003b). The species dwells in both open and closed habitats (von Proschwitz 2003), including *Glyceria maxima* swamps and sedge marshes, fens, reed (*Phragmites australis*) swamps, riparian margins, as well as alder cars (*Alnus glutinosa* woodlands) densely vegetated by *Carex* ssp. or *Iris* (Killeen 2003a,b).

Since habitats of *V. moulinsiana* are flooded periodically, the snail climbs up emergent vegetation, reaching a height of over 2 m above ground (Killeen 2003b). However, these vertical migrations are age-dependent and show a periodicity (e.g. Książkiewicz-Parulska 2020). Juveniles are usually found on the ground, within the leaf litter, while adults occur on above-ground vegetation (e.g. Książkiewcz-Parulska and Gołdyn 2017). Such microhabitat segregation of different age stages is probably related with age-dependent requirements for humidity and nutrition (Książkiewicz-Parulska 2020). The litter in wetland habitats is moist, shaded and cooler than the plant parts above the ground, and also contains abundant and diverse sources of food (Bondesen 1966). The above-ground stems and leaves of monocotyledonous plants are drier, hotter and more exposed (Geiger 1965), but may offer protection from flooding (Książkiewicz-Parulska 2019, 2020), from invertebrate predation (McKemey et al. 2001; Hasegawa et al. 2009), and from parasitism (McCoy and Nudds 1997; Podroužková 2015). In contrast, adults are abundant on plants from the beginning of summer, during autumn, and winter, whereas in springtime the snails descend towards the litter. At the beginning of growing seasons, *V. moulinsiana* starts to reproduce, so the downward migration to the litter is related to oviposition behaviour (Książkiewicz-Parulska 2019, 2020).

## Results

### Molecular identification of the parasite

DNA-barcoding based on sequencing of the mitochondrial *COI* gene fragment showed that the detected parasite belongs to the dipteran species *Limnia unguicornis*. The *COI* sequence variant found in this study (GenBank acc. no. PQ444201) showed 99% identity with the COI sequence found in *L. unguicornis* voucher MZH_HP.852 mitochondrial genome (GenBank acc. no. MZ607737).

**Fig. 1:**
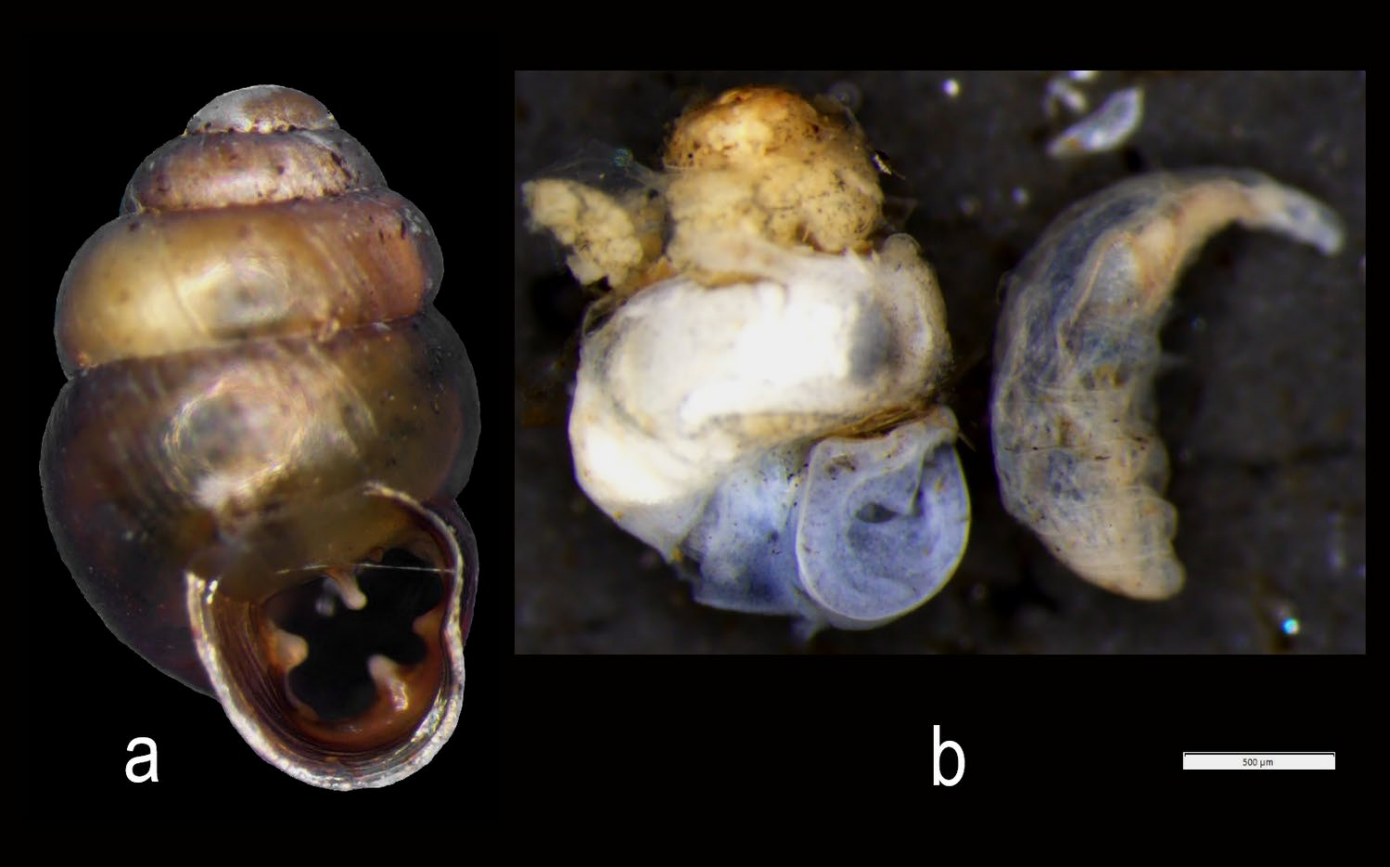
Shell of *Vertigo moulinsiana* before dissection (**a**) and body of snail with *Limnia* larva after dissection (**b**). Scale preserved

This result indicates that in this paper we report for the first time a larva of *L. unguicornis* parasitic on *Vertigo moulinsiana*. This way, we have enriched the list of dipteran larvae parasiting on *Vertigo* species. The larva of *L. unguicornis* was found in one out of the 30 individuals examined from the study site. The dipteran larva was 1.4 mm long, was completely hidden in the shell of *V. moulinsiana*, and not visible during the external examination of the snail (Fig. 1a).

**Fig. 2:**
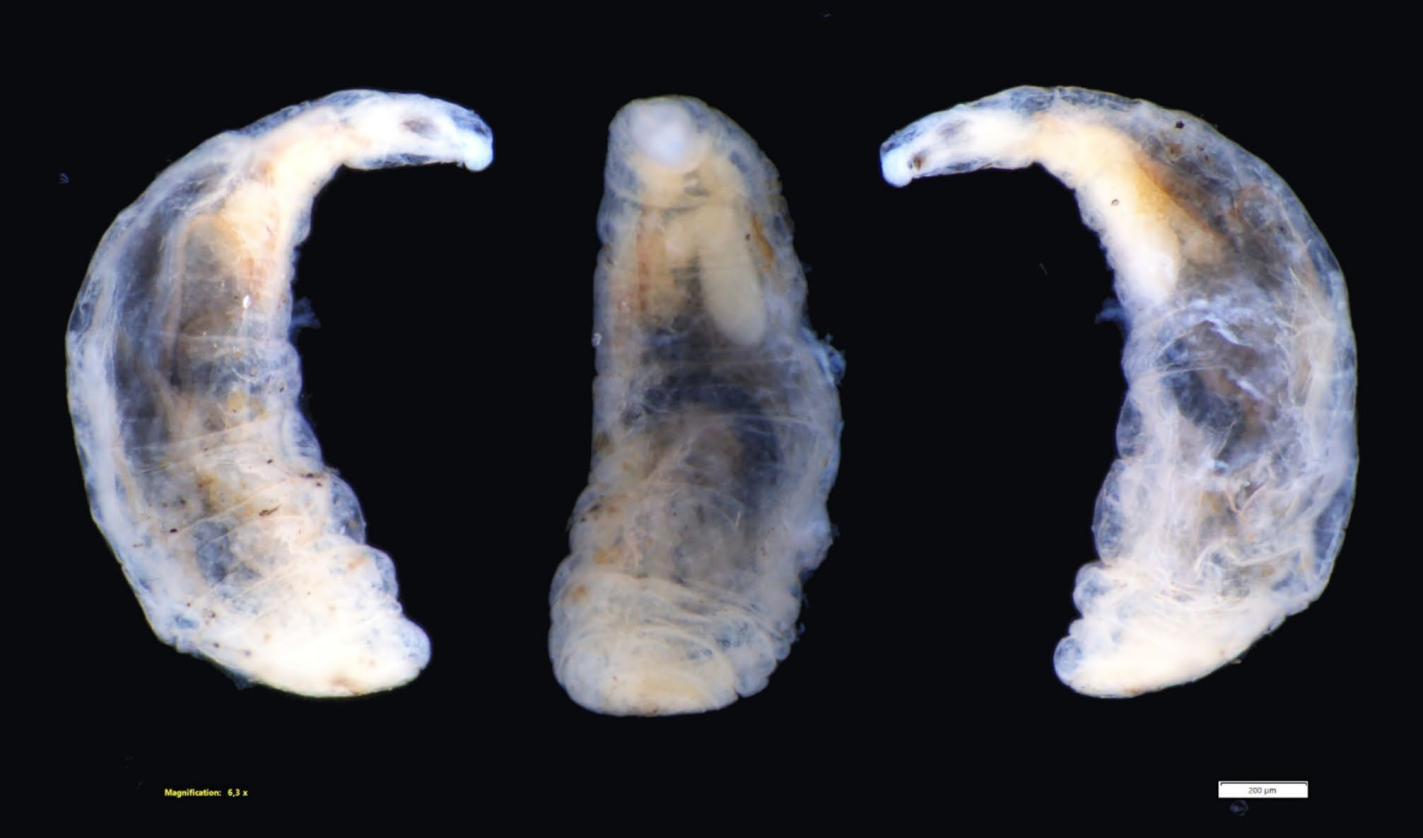
Body of the *Limnia* larva, view from left: lateral right, ventral, and lateral left views

After separating the shell from the soft tissues of the snail, the larva was found (Fig. 1b, Fig. 2). Its body occupied a whole whorl of the shell. The final section of the dipteran abdomen was located about ¼ whorl before the shell’s aperture. Considering the larval body flexibility, as well as the presence of a constricted rostrum, the parasite may potentially penetrate the shell deeper than we found during the examination. We also discovered that the snail’s body was tightly retracted and occupied less than half of the shell (beginning from the shell apex) (Fig. 1b). The snail body (if free from the parasite) usually occupies almost the whole shell or is gently retracted within the last whorl (personal observation).

## Discussion

The parasitic relations between the Diptera (including the *Sciomyzidae*) and representatives of the snail family *Vertiginidae* have already been mentioned in the literature. For example, an unknown larva was found to share the shell with *Vertigo genesi* in 1937 by Bhatia and Keilin (1937). On the basis of their report, Foote (1996) recognized that the larva belonged to the Tetanocerini, most likely to the genus Tetanocera. Over 25 years later, Williams et al. (2023) identified this species as *Trypetoptera punctulata* (Scopoli, 1763). Recent studies have shown that *T. punctulata* may also feed on other vertiginids including the wetland species *Vertigo antivertigo* (Gaponov 2016). Moreover, in a shell of *V. antivertigo* also a larva of *Tetanura pallidiventris* (Fallen, 1820) was found (Gaponov 2016).

A laboratory study of Vala and Knutson (1990) indicates that *Limnia unguicornis* larvae, when hatching from eggs, are about 1-mm long and the first larva may reach up to 3 mm in length. Thus, the dipteran we found within the shell of *Vertigo moulinsiana* is most probably the first larval stage (as mentioned before, the larva we found was 1.4-mm long). The condition of the snail’s soft tissues indicates that the larva was still alive when collected and preserved. Moreover, the vertiginid body seemed to be intact: no tissues were missing and no feeding marks were found. This observation is consistent with the cited study, according to which the two first larval stages are parasitic and feed mainly on blood.

Considering the study of Vala and Knutson (1990), we suppose that *V. moulinsiana* is too small to provide shelter and food for the entire larval developmental period of *L. unguicornis*. As it was already mentioned, switching the host is possible but rare according to the cited authors. Comparing the size of the host (whorls and aperture diameter) and the parasitic larva, we presume that host-swapping would be needed at the second larval stage. Since the larvae usually complete development utilizing one host only (Vala and Knutson 1990), it is puzzling why *L. unguicornis* chose as a host the snail species which seems to be too small for the larva to complete its development. Perhaps the time the larva spent on searching for a host, was long enough to expose it to energy loss, desiccation, and predation. Supposedly due to a lack of a proper host, the larva attacked the first snail that came along, just to save itself from death. *Vertigo moulinsiana* is a climbing species, living on monocot plants. The climbing behaviour is also found in *Succinea* species, which are usually chosen by larvae of *L. unguicornis* for a host. However, this hypothetical scenario needs to be supported by a more detailed study assessing if parasitism on *V. moulinsiana* was rare, accidental event or is it more common.

Concluding, this paper presents the first record of parasitism involving a dipteran larva from the genus *Limnia* and snail from the family *Vertiginidae*. Considering the previous studies, according to which *Limnia* larvae complete development within one host, it is baffling that the larva attacked an insufficiently big snail species. On the other hand, selecting the minute snail may be beneficial at the first larval stage. The smaller host may be easier to be attacked than a bigger one, and also weaker, preventing the larva from being pushed out of the shell. Switching the host to a bigger one at the second larval stage (when it is stronger), although exposing the dipteran parasite to desiccation and predation after leaving the snail shell, may involve a greater likelihood of success and bring stability within the next host-snail. Thus, our observation indicates that the *Limnia* larvae may demonstrate more complicated strategies than it was suggested by Vala and Knutson (1990) (i.e. entire larval developmental period within a one host). The data we have are too limited to determine the frequency of parasitism of *Limnia* larvae on *Vertigo moulinsiana* (because only one population was studied during one sampling event). Further research is needed to assess if it is a common relation, and how it affects the threatened *Vertigo* species population.

## Supplementary Information

Below is the link to the electronic supplementary material.

Supplementary file1 (DOCX 68 KB)

## Data Availability

No datasets were generated or analysed during the current study.
