# Supplementary material for: The first record the Limnia unguicornis (Diptera, Sciomyzidae) parasites on a vulnerable pulmonate land snail, Vertigo moulinsiana (Gastropoda: Eupulmonata: Vertiginidae) and a literature review on Limnia species
Source: Parasitol Res. 2024 Nov 1;123(11):367. doi: 10.1007/s00436-024-08388-7 (PMC11527932; doi:10.1007/s00436-024-08388-7)
Supplement: Supplementary file 1 — Supplementary file1 (DOCX 68 KB) [file 436_2024_8388_MOESM1_ESM.docx]

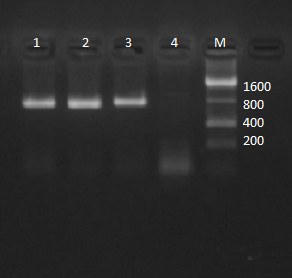


Photograph of a gel prior to NGS sequencing showing the PCR product containing the NGS adapter sequence, index, primer and insert (1. PCR product [not diluted]; 2. PCR product [2x diluted]; 3. positive control; 4. negative control; M - size marker).
